# Nanocharacterization in Dentistry

**DOI:** 10.3390/ijms11062523

**Published:** 2010-06-17

**Authors:** Shivani Sharma, Sarah E. Cross, Carlin Hsueh, Ruseen P. Wali, Adam Z. Stieg, James K. Gimzewski

**Affiliations:** 1 Department of Chemistry and Biochemistry, University of California, Los Angeles 90095, CA, USA; E-Mails: sharmas@ucla.edu (S.S.); secross@gmail.com (S.E.C.); carlin.hsueh@gmail.com (C.H.); rpwali@chem.ucla.edu (R.P.W.); 2 California NanoSystems Institute, University of California, Los Angeles 90095, CA, USA; E-Mail: stieg@cnsi.ucla.edu (A.Z.S.); 3 International Center for Materials Nanoarchitectonics Satellite (MANA), National Institute for Materials Science (NIMS), Tsukuba 305-0047, Japan

**Keywords:** nano-characterization, dentistry, biofilms, bacterial adhesins, implants, dentine tubule, afm, interferometry, nanodentistry

## Abstract

About 80% of US adults have some form of dental disease. There are a variety of new dental products available, ranging from implants to oral hygiene products that rely on nanoscale properties. Here, the application of AFM (Atomic Force Microscopy) and optical interferometry to a range of dentistry issues, including characterization of dental enamel, oral bacteria, biofilms and the role of surface proteins in biochemical and nanomechanical properties of bacterial adhesins, is reviewed. We also include studies of new products blocking dentine tubules to alleviate hypersensitivity; antimicrobial effects of mouthwash and characterizing nanoparticle coated dental implants. An outlook on future “nanodentistry” developments such as saliva exosomes based diagnostics, designing biocompatible, antimicrobial dental implants and personalized dental healthcare is presented.

## 1. Introduction

**Figure 1:**
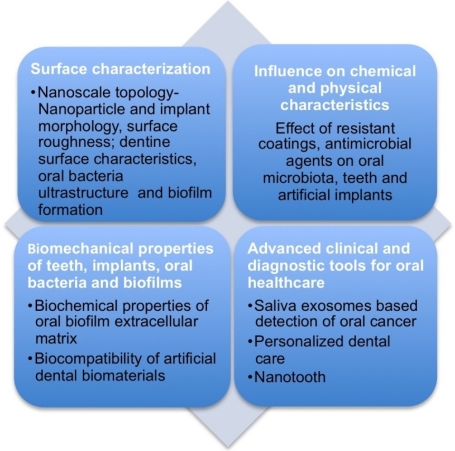
Atomic force microscopy (AFM) based nanoscale topographic, biophysical and biochemical characteristics of dental surfaces and structures.

Emerging technologies and new nanoscale information have the potential to transform dental practice [1] by advancing all aspects of dental diagnostics, therapeutics and cosmetic dentistry [2] into a new paradigm of state-of-the-art patient care beyond traditional oral care methods and procedures. One of the key changes is the application of new research tools that have changed the size-scale of dental research. Nanotechnology permits a new understanding and manipulation of these biological processes and materials at the nanoscale (1–100 nm) level. Based on their unique capabilities and resolution, nanoscale science probes surfaces using forces, displacement resolutions and concentrations on the piconewton, nanometer and picomolar scales, respectively. Studying dental structures and surfaces from a nanoscale perspective may lead to better understanding of the structure-function-physiological relationship of dental surfaces. Using nanocharacterization tools, a variety of oral diseases can be understood at the molecular and cellular levels and thereby prevented. Nano-enabled technologies thus provides an alternative and superior approach to assess the onset or progression of diseases, to identify targets for treatment interventions as well as the ability to design more biocompatible, microbe resistant dental materials and implants. Quantitative nanoscale topology, biophysical and biochemical nano and microscale properties of dental surfaces and implants are thus of significant interest in a wide array of dental research and development. Figure 1 describes some of the extensive applications of Atomic force microscopy (AFM) to quantitatively probe the topographic, mechanical and biochemical properties of various biotic and artificial dental surfaces and structures. There exists a huge body of excellent work, spanning more than 15–20 years on bacterial ultrastructure AFM imaging [3–7], *in vivo* force spectroscopy on bacteria [8–10] and bacterial nanomechanical characterization [11–14]. Here, we discuss the role of AFM and optical interferometry as nano-characterization tools in dentistry.

## 2. Nanoscale Observation of Dentine Surfaces and Collagen Network

**Figure 2:**
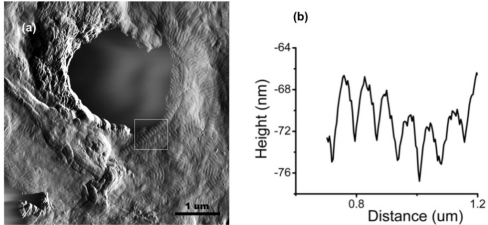
(**a**) Inverted AFM amplitude image of untreated dentine surface with a single open tubule and surrounding exposed collagen network. White box marks periodic banding of ∼67 nm indicative of collagen D-banding. (**b**) Sectional profile of the collagen fibril. Reprinted with permission from [19]. Copyright 2009Yes Group, Inc.

Dental pain such as toothache is a commonly experienced problem and a major reason why patients visit the dentist, thus making the study of tooth enamel, dentine, and its collagen network a necessary endeavor. Microscopic tubes, called dentine tubules, run through dentine, from the pulp beneath, to the junction with the enamel above, down to the nerves. A major component of the organic material in dentine and bone is collagen [15]. Observing the dentine surface structure and collagen networks at the nanoscope scale can help improve restorative and regenerative dentistry, collagen based materials in tissue engineering, and our understanding of disease processes related to bone weakness, such as osteoporosis [16]. AFM is useful in studies of the collagen network and dentine surface changes caused by different chemical agents [17,18] because of minimal sample modification and alteration needed before and during scanning, unlike the conducting gold or carbon coating required when using scanning electron microscopy (SEM). SEM also requires a certain degree of sample electrical conductivity and works in a vacuum environment. Figure 2 shows a typical AFM scan of dentine tubules on the surface of a human tooth revealing helical structures observed in untreated enamel samples [19]. These densely packed fibril structures on the surface of the dentine have a banded periodicity averaging ∼67 nm, agreeing with previous studies on collagen “D-Banding” structures [16]. Figure 2(a) shows an AFM amplitude image of an untreated sample with a visibly exposed collagen network over the majority of the surface. Figure 2(b) shows sectional analysis of the periodic banding that is indicative of collagen structure. This banding is consistent throughout the surface. AFM based structural analysis of dentine and of its collagen components may be critical in understanding the structure of native, fully mineralized, skeletal substrates. This will allow us to investigate the material and morphological properties of dental and other connective tissues such as bone *in situ* and enable early discrimination of various patho-physiological states and disease progression such as osteoporosis where skeletal tissue is mechanically weakened [20].

## 3. Dentine Tubules Blocking – to Alleviate Hypersensitivity

Dentine hypersensitivity is an acute pain condition that typically occurs when the surface of the root becomes exposed. When the gingiva has receded and cementum removed, the dentine tubules become exposed and opened and then fluid flow along these open tubules caused by mechanical, chemical or thermal stimuli can result in an uncomfortable pain response in the nerve fibers [21]. Among the many approaches [22] to treating dentine hypersensitivity, one primary approach is occluding dentine tubules; open tubules are sealed and isolated from external stimuli, preventing fluid movement from triggering a pain response [23]. AFM has recently been used to observe the effective occlusion of dentine tubules with a new arginine-calcium carbonate technology developed for treating sensitive teeth [19,23]. A desensitizing prophylaxis paste (marketed in the United States as Colgate Sensitive Pro-Relief Desensitizing Paste, Colgate Palmolive, USA) with 8% arginine, calcium carbonate, and prophylaxis-grade silica, has been clinically proven to effectively plug and seal dentine tubules [19,24].

**Figure 3:**
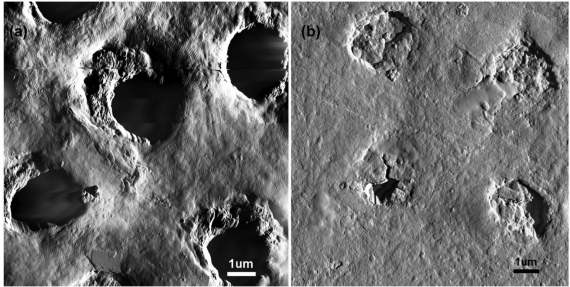
(**a**) Inverted AFM amplitude images of untreated dentine surface with exposed and opened tubules. Visible helical structures can be seen on surface. (**b**) Dentine surface after ProClude prophylaxis paste treatment. Helical structures are absent from surface and tubules are occluded, suggesting the treatment resulted in a protective layer over the exposed dentine surface and sealing of the open tubules. Reprinted with permission from [19]. Copyright 2009Yes Group, Inc.

It is important to gain insight into the mechanism of action of this new technology for dentine hypersensitivity relief, especially regarding the nature and extent of dentine tubule occlusion. AFM was used to image the surface of dentine samples (Figure 3) with open tubules, and those that have been occluded by treatment five times with the desensitizing prophylaxis paste [19]. Untreated dentine surfaces show open tubules with helical fine structure on the dentine surface from the exposed collagen network of the tooth structure. Treated specimens (Figure 3(b)) do not show surface helical structure as a result of the formation of a protective layer and sealing of the tubules. High- resolution AFM images showing nanometer resolution of the dentine surface confirm that this new arginine-calcium carbonate technology is highly effective in occluding dentine tubules.

Thus, AFM has shown to be a useful tool for the study of dentine surfaces and collagenous tissues that indicates its potential in understanding oral disease processes. Alternate modes of AFM also prove to be useful in studying dental surfaces, such as piezoresponse force microscopy (PFM) to differentiate between organic and mineral components on dental tissues with nanoscale resolution [25]. Further research utilizing AFM’s ability to simultaneously collect qualitative and quantitative analysis of dentine and collagen at the nanoscale should therefore prove essential in providing important insights on the effectiveness of oral treatments for periodontal disease prevention [26], disease progression, and development in collagen dependant materials such as bone, cartilage, tendons, skin, collagen-based materials in tissue engineering and biomedical device coating [16].

## 4. Nanocharacterization of Cell-Surface Macromolecules and Cell-Wall-Associated Proteins Native to *Streptococcus mutans*

Bacteria colonize the oral cavity surfaces through assembling multicellular communities via cell-cell and cell-surface interactions [27]. With the formation of biofilms such as those produced by *S. mutans* (commonly referred to as dental plaque), cell-surface interactions play an enormous role in initial cell colonization and cell-cell protein interactions largely mediate biofilm growth and continued existence.

The function, structure and properties of bacterial cell-surfaces are determined by the presence of species-specific proteins, lipids, and polysaccharides. Lactic acid producing bacteria like *Streptococcusmutans*, *Streptococcus sobrinus* and *Leuconostocmesenteroides* are known to produce specific exopolysaccharides called glucans [28,29]. One of the most important virulence factors of *S. mutans* is the synthesis of glucan from sucrose [30]. Glucan synthesis allows the bacteria to firmly attach to the tooth surface and form a biofilm, while the gelatinous nature of glucan retards diffusion of acid produced by the bacteria from fermentable sugars in the dental plaque [31]. This eventually leads to dissolution of the hard enamel surface of the tooth and cavity formation. Research on dental plaque development and the etiology of dental caries established the central role of glucans in sucrose-dependent adhesion and the correlation between sucrose consumption and increased caries rates [32,33]. In *S. mutans,* glucans are synthesized from sucrose by the enzymatic action of three types of glucosyltransferases (Gtfs): GtfB and GtfC synthesize mainly water-insoluble glucans (>85%) with α(1–3) glucosidic bonds (mutan); GtfD forms water-soluble glucans (>70%) with α(1–6) glucosidic bonds (dextran) [34]. The contribution of the individual glucans to cariogenicity has been the focus of many studies in the past [35–37].

*In vivo* techniques such as AFM provide a novel, nondestructive method for providing insight into critical properties associated with bacterial cells and their related surface proteins. AFM has proved to be a powerful tool for not only imaging bacterial ultra-structure surfaces under *in situ* conditions, but also for determining the associated mechanical properties and intermolecular forces [38,39]. Schär-Zammaretti and Ubbink probed the surface properties of different *Lactobacillus* strains using AFM to quantify tip-cell-surface adhesion forces and related this study to the ability of the Lactobacilli to adhere to surfaces, clustering, auto- and co-aggregation [40,41]. van der Mei *et al*. used AFM to determine cellular stiffness of fibrillated and non-fibrillated strains of *Streptococcus salivarius* [42]. These studies demonstrate the use of AFM for characterization of bacterial surface properties under physiological conditions, which have resulted in a greater understanding of bacterial structure and function [37].

### *Exploring Cell-Surface Interactions: Analysis of Local Cell-Surface Interactions Associated with the Glucan Polymers of* S. mutans *Using* in Situ *Force Spectroscopy*

**Figure 4:**
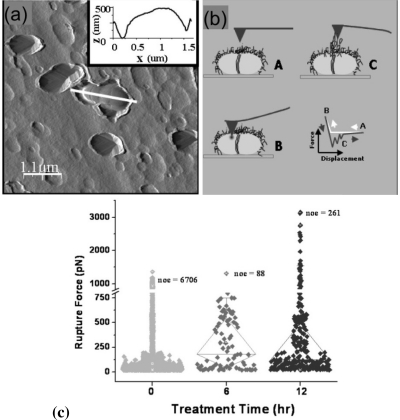
(**a**) AFM deflection mode image of a mechanically trapped *S. mutans* wild-type cell in fluid. The inset shows the height profile corresponding to the white line drawn along the long axis of the mechanically trapped cell. (**b**) Schematic representation of an AFM tip interacting with cell-surface macromolecules; A = Before tip-cell interaction; B = Tip pushing into cell surface; C = Tip pulling away from cell surface. The force-displacement curve shows typical tip-cell interactions. (**c**) Rupture force measured between the AFM tip and *S. mutans* UA140 wild-type cells at treatment time (0, 6, and 12 h). The average rupture force for the wild-type cells in each case, untreated control (0 h), 6 h and 12 h sucrose-treated is 84 ± 156, 304 ± 282 and 376 ± 563 pN, respectively (P < 0.00001 for control *vs.* both 6 h and 12 h) from n = 100 curves done on three individual cells in each case. Reprinted with permission from [37]. Copyright 2007 Society for General Microbiology.

Individual strains of *S. mutans* probed using AFM to determine the role of glucans in cell adhesion and aggregation of *S. mutans* (Figure 4), yield quantitative information regarding the mechanics of these glucan-polymer macromolecules [37].

Cross *et al.* report results for 1% sucrose treated cells compared to control (*ctrl*), untreated cells [37]. To determine the right time point for probing of *S. mutans* cells with the AFM, cells grown without sucrose were compared to those supplemented with 1% sucrose in the exponential growth phase and cells which reached the stationary phase. *S. mutans* wild-type untreated (*ctrl*) cells yielded adhesion forces for the observed rupture events ranging from ≈20–330 pN (Figure 4). After growth of these cells with sucrose for 6 h tip-cell adhesion forces increased in range to ≈20–1310 pN and after growth for 12 h the required unbinding force increased dramatically to range between ≈20 pN and 3150 pN (Figure 4). The mean rupture force for *S. mutans* UA140 wild-type *ctrl*, 6 h and 12 h sucrose treated cells was 84.1 ± 156.0, 304.3 ± 281.9, and 375.6 ± 563.2, respectively. It was found that the population mean for *S. mutans* UA140 wild-type *ctrl* cells was significantly different from both the population means for the 6 h and 12 h sucrose treated conditions (P ≥ 0.00001). However, the population means for *S. mutans* UA140 wild-type cells after 6 h and 12 h of sucrose treatment were not significantly different. Additionally, the large number of unbinding events observed in the case of the wt *ctrl* cells is most likely due to the presence of several surface proteins, such as Pac and Wap [43,44], which under non-sucrose conditions may cause increased tip-cell-surface interactions, whereas after sucrose is introduced into the system glucan chain growth induces tip-cellsurface protein interactions at a tip-cell distance significantly larger than that required for tip interactions with shorter surface proteins, thus yielding less overall adhesion events yet much stronger binding events. The effect of sucrose was also examined on five different *S. mutans* UA140 mutant strains, which had individual mutations in specific *gtf* genes [37]. AFM force-displacement curves collected on *S. mutans* UA140 *gtfB*, *gtfC*, *gtfD*, *gtfBC* and *gtfBCD* mutant strains revealed specific biochemical and biophysical properties of microbial surfaces resulting from the enzymatic activity of *Gtfs* at the cellular level [37]. The results indicate that the optimum adhesion for *S. mutan s*cells occurs in the stationary phase, and sucrose is most likely used up by the cells for the synthesis of the glucan polymers and metabolic purposes.

## 5. The Effect of *wapA* Mutation on Cell Surface Adhesion in *S. mutans*

In addition to sucrose-dependent biofilm formation, the mechanism of sucrose-independent surface attachment is another important factor in biofilm formation and aggregation. In the absence of sucrose, the production of particular cell-wall-associated proteins becomes important for successful colonization of *S. mutans* in the oral cavity [45]. The surface-associated protein Antigen I/II (Pac), which binds to the salivary glycoproteins, has been shown to be required for the initial attachment of *S. mutans* to the saliva coated tooth surface [44]. LytR, a homolog of a regulator of autolysin activity in *Bacillus subtilis,* was shown to play an important role in sucrose-independent attachment to polystyrene surfaces in *S. mutans* [46,47]. Another surface associated protein, WapA, is a well-studied human vaccine candidate [43,48]. To elucidate the structural and biological functions of WapA in *S. mutans*, Zhu *et al.* [45] used AFM along with other methods to show that WapA is involved in sucrose-independent cell-cell aggregation and biofilm formation.

**Figure 5:**
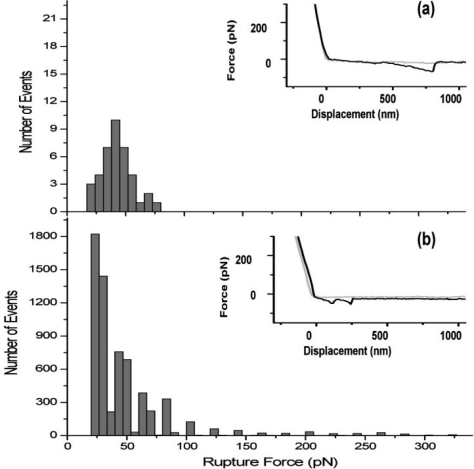
Histograms of rupture force between the AFM tip and living *S. mutans* wapA mutant (**a**) and wild type (**b**) cells. Insets show typical force-displacement curves revealing adhesive interactions between the cantilever tip and cell surface in the retract trace. In the case of *S. mutans* wild type cells (b), adhesion forces for the observed rupture events ranged from ≈20–330 pN. Those observed for the wapA mutant cells (a) had a range between ≈20–80 pN. The average rupture force for the wapA mutant and UA140 wild type cells is 43 ± 13 pN and 84 ± 156 pN, respectively. Reprinted in part with permission from [45]. Copyright 2007 Society for General Microbiology.

*In vivo* force spectroscopy of living *S. mutans* wild type and *wapA* mutant cells revealed the cell adhesion as a function of the nanomechanical properties of the existing surface adhesive molecules [45]. Adhesion forces for living *S. mutans* wild type UA140 and *wapA* mutant cells were obtained, revealing average rupture forces of 84 ± 156 pN and 42 ± 13 pN, respectively. Probed under analogous conditions, the wild type cells exhibited considerably more adhesion events as compared to the mutant cells. In particular, wild type (Figure 5(b) inset) cells revealed sawtooth-like patterns in the retraction traces of the force-displacement curves indicative of multiple tip-cell interactions, whereas *wapA* mutant (Figure 5(a) inset) cells in general only revealed single tip-cell adhesion events. As shown by the histogram in Figure 5(a), the rupture forces observed due to tip-cell adhesion fall within a much narrower range of ≈20–80 pN for the *wapA* mutant cells as compared to the wild type cells, whose rupture events range from ≈20–330 pN (Figure 5(b)). Moreover, the number of rupture events observed for the wild type cells is significantly more than the number of events exhibited by the mutant cells. Since these analyses were conducted for the wild type and mutant cells under the same conditions, these results suggest that the wild type cells are more adhesive than the *wapA* mutant cells. The contribution of the individual glucan-polymers and wall-associated proteins to molecular level interfacial properties can thus be probed using AFM, a technique which has become pivotal for imaging of bacterial ultra-structure surfaces under *in vivo* and *in situ* conditions and for determining mechanical properties and molecular forces of bacterial cell [40–42,49,50]. Using AFM based force spectroscopy, the mechanics associated with particular cell-surface macromolecules, such as glucans, for *S. mutans* UA140 wild-type and isogenic mutant strains can be quantified under physiological conditions [37].

## 6. Dental Plaque-Antimicrobial Agents for Prevention of Bacterial Biofilms

Bacteria are the primary etiologic agents in periodontal disease. It is now well recognized that dental plaque is predominantly a complex bacterial biofilm. These diverse bacterial species have evolved to inhabit the environment of the tooth surface, gingival epithelium, and oral cavity since birth. Amongst these, *Streptococcus mutans* is widely considered to be the principal pathogen responsible for dental caries [30], one of the most prevalent infectious diseases afflicting humans [51]. They form a well-organized community of bacteria that adheres strongly to dental surfaces and are embedded within an extracellular polysaccharide containing slime layer. *S. mutans* biofilms are usually more resistant to antimicrobial agents than planktonic organisms, as they are encased in the extracellular matrix thereby impeding access of the agent to the bacteria and because the phenotypic changes themselves may render the bacteria more resistant [52].

**Figure 6:**
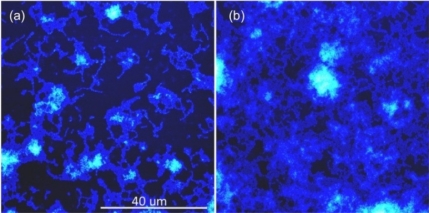
(**a**) AFM height images showing topography of listerine treated *S.mutans* biofilm. (**b**) Untreated *S. mutans* biofilm topography showing clustered microcolonies. The peak biofilm height decreased from ∼1 μm to <0.5 μm when the biofilms were treated with mouthwash.

**Figure 7:**
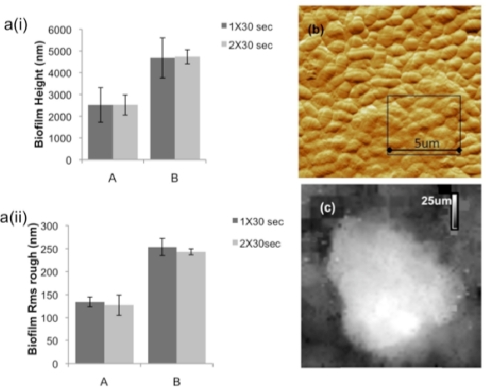
(**a**) Comparison of *S. Mutans* (A) mouthwash treated and (B) untreated biofilm peak height (i) and surface roughness (ii) for 1 × 30 s and 2 × 30 s treatment. (**b**) High resolution AFM topographic image showing rough EPS matrix of the biofilm visble as granular matrix surrounding the bacterial cell surfaces. (**c**) Scanning Ion Conductance Microscopy enables imaging larger scan area under physiological conditions. 64 × 64 μm^2^ *S. mutans* biofilm topography is shown here.

However, much of the dental disease conditions, including dental caries, can be prevented by a simple yet effective measure of thorough daily control of dental plaque [53]. A wide range of clinical studies has been presented over the years to develop effective strategies to prevent and control periodontitis, such as the effectiveness of therapeutic antimicrobial mouth rinses [54]. Studying bacterial biofilms is relevant to antimicrobial mouth rinse studies, as it enables critical assessments of its effect against the plaque biofilm under actual-use conditions rather than on less resistant planktonic organisms that may not be indicative of the mouth rinses effectiveness [53]. Visualization of bacterial cell surface architecture at nanoscale resolution and quantitative studies on biochemical and adhesion properties of bacterial biofilms using AFM provide unique data not measurable by standard optical microscopy [50,55]. The major advantages of AFM includes its ability to “zoom” in and out over the magnification range of both optical and electron microscopies, but under natural imaging conditions in liquid with minimal to no sample preparation, and can produce real-space quantifiable three-dimensional images of the surfaces. AFM has been successfully applied to investigate nanometer-scale topographical changes resulting from the treatment of *S. mutans* biofilms to various mouth rinse treatments (Figure 6(a)). Height images and roughness analyses were performed on three days old biofilms grown on glass cover slips and subjected to 1 × 30 or 2 × 30 s treatments of Listerine to assess its effectiveness towards plaque management by characterizing the changing topography and surface characteristics of *S. Mutans* biofilms (Figure 7(a)).

Surface roughness analysis allows quantification of extracellular polymeric slime (EPS) content for different treated biofilms of *S. mutans* (Figure 7(b)). Surface roughness values (Rms) for the extracellular matrix of the biofilms can be calculated from high-resolution AFM topographic images. For each biofilm sample, the average *R*rms value was calculated by collecting the *R*rms values for two 5 × 5 μm^2^ areas at the highest peak regions within the biofilm structure (Figure 7(c)). The effectiveness of mouth rinse solution on *S. mutans* biofilms was analyzed based on the ability to decrease biofilm height (topographic changes) and lower biofilm surface roughness (corresponding to EPS matrix disruption). Comparison of *S. mutans* biofilm peak height and surface roughness show about two-fold decrease in the mouthwash treated biofilms compared to untreated control. So far, AFM is the major nanotechnology technique in use for analyses of cells and biofilm surfaces and can provide exquisite topographic imaging, coupled with detailed microphysical and nanophysical probing and characterization of biofilm surfaces. An array of related scanning probe microscopy (SPM) technologies, including scanning ion conductance microscopy (SICM) [56] further expand the characterization capabilities of this family of non-photonic imaging technologies. Most AFMs have a z range up to few microns that limits the imaging of surfaces such as mature biofilms, which are several microns rough at the surface and thus challenging to image via AFM. Figure 7(c) provides an example of the vertical and lateral resolution possible with SICM approach. Furthermore, correlation approaches also enable parallel characterization of biofilm surfaces using both light or fluorescence microscopy and AFM, expanding analytical capability and depth. Using this approach, it is possible to rapidly test and develop new advances in formulation based on the various factors such as geographic location that are used in developing these highly sophisticated oral hygiene products.

## 7. Dental Implants: Structure, Chemistry and Biocompatibility

Dental implantology has a long, well documented history reaching back over thousands of years from ancient times and initial modern reports in the early 19th century to the accidental discovery of osseointegration by Brånemark in 1952 and its subsequent acceptance in the dental community some 25 years later. As defined, osseointegration refers to non-mechanical anchorage or retention of a load-bearing alloplastic material through direct structural attachment to osseus tissue without the need for intervening connective tissue [57,58]. In contrast to a simple mechanical approach where surface contact area and topography are the sole deterministic factors, bioactive approaches such as osseointegration involve the direct physiochemical bond formation and most commonly involve the use of titanium implants or alloys thereof in combination with hydroxyapatite surface functionalities.

A moderately roughened microscale topography and calcium phosphate mineralization of osseus tissue surfaces have been shown to be two critical features in the successful osseointegration of implant materials [59,60]. While extensive research on the effects and subsequent optimization of microtopography and surface chemistry has produced ground-breaking strides in materials engineering such as the widely used Osseotite® dental implant (BIOMET 3i, Palm Beach Garden, FL), the capacity provided by advances in nanoscience and nanotechnology to generate as well as characterize variations in both features at the nanoscale has received far less attention [61–63]. As a result, differentiation of those material properties that enhance osseointegration, identification of potential synergistic effects, as well as unambiguous interpretation of long-term clinical datasets remains a challenge for researchers, manufacturers and clinicians.

**Figure 8:**
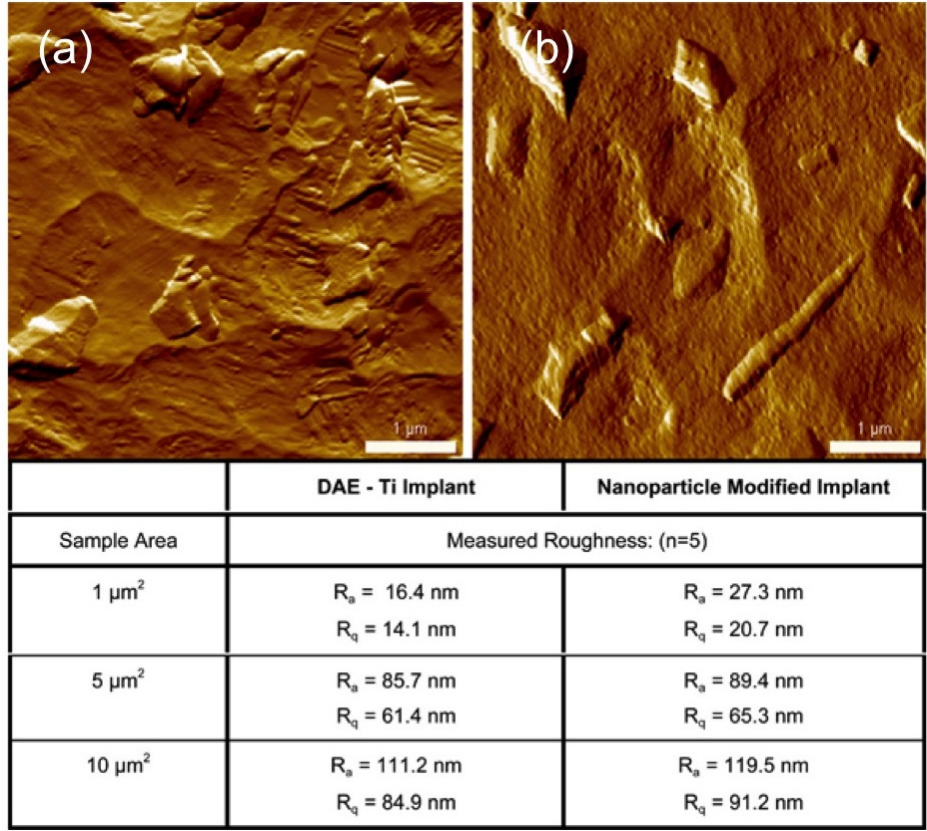
(**a**) AFM height images and surface roughness analysis showing variations in nanoscale topography between dual-acid-etched (DAE) titanium implant surface titanium before deposition of discrete hydroxyapatitenanoparticles. (**b**) After deposition of hydroxyapatitenanoparticles.

A simple example of the relevance in using advanced nanoscale characterization techniques in such systems involves the use of hydroxyapatite surface modifications and their subsequent effects with respect to biocompatibility and osseointegration. Despite the clear rationale for the design and use of such materials, initial applications produced strong negative results with high failure rates that were ultimately attributed to mechanical effects [64]. Subsequent studies using nanoscale approaches to modified surface chemistry in combination with multi-scale topographic variation have demonstrated nanotopography to be of greater importance than the substrate chemistry, high levels of osseointegration both *in vitro* and *in vivo* and favorable biocompatibility [65]. In such cases, an ability to characterize nanoscale topography quantitatively has been shown to be critical towards elucidation of underlying physiochemical mechanisms. Figure 8 provides an example of such characterization by AFM where analyses carried out over relatively large areas (≥5 μm^2^) show little variation on surface roughness. However, at sub-micron length scales, substantial variation in nanoscale topography can be observed. In addition to imaging, AFM also provides the opportunity to detect the fundamental forces acting between surfaces, molecules and even cells through force spectroscopy. Future application of such highly sensitive, nanoscale characterization tools to the field of dental implantology will undoubtedly serve to more fully elucidate the functional physiochemical mechanisms at play.

## 8. Bacteria-Induced Enamel Demineralization Studied Using Optical Profilometry

Historically, dental profilometry has been performed with a thin stylus about 20 microns in tip diameter that is dragged slowly across a surface at a rate of 10 mm/min to yield an image [54]. In a typical study Hughes *et al.* looked at the effect of pH and concentration of acids common in foods on enamel erosion rates [55]. In the past two decades, this technique has been combined with computer graphics software to provide 3D images of the surface. Tantbirojn *et al.* used profilometry to map stresses in dental restorations through deformation [56]. The stylus is made of a rigid material like metal or diamond and with sensitive samples, care must be taken not to scratch the surface. Alternative non-destructive methods have emerged and begun to compete with stylus-based profilometry. Recently, optical profilometry has been used in dental analysis as well. Barbour and colleagues looked at the softening of enamel from exposure to soft drinks, performing hardness tests with indentations imaged using an optical profiler [57]. Zhang and coworkers tracked enamel demineralization using the surface roughness from an optical profile as the metric [58]. Typical optical profilometers have vertical resolution of a few nm and lateral resolution of a few microns [59]. Optical profiling enables measurement of tooth enamel decay with vertical resolution under one-nanometer and lateral features with optical resolution [66]. Optical profilometry is non-destructive, using white light at relatively low power, and square measurements hundreds of microns on a sample can be made in a few seconds [67]. Reflected light from the sample surface is mixed with the original beam to create an interference pattern and generate a 3D surface structure. The technique offers the capabilities and flexibility of lateral optical resolution as in optical microscopy with nanometerscale height resolution and the ability to perform dynamic studies in real-time in liquid environments [68,69].

**Figure 9:**
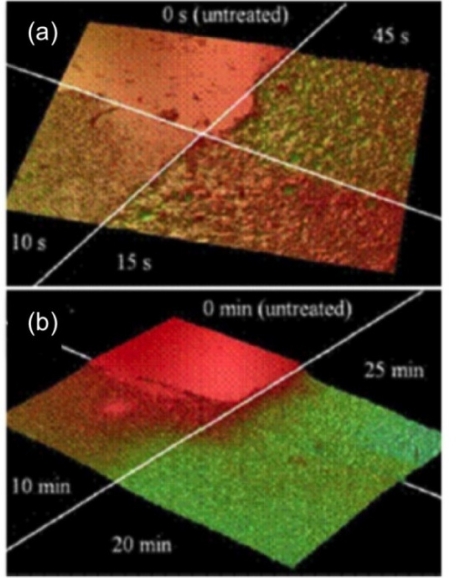
Optical Profiler analysis of citric acid induced dental enamel erosion analysis. (**a**) An image of four quadrants of dental enamel eroded for various times less than one minute. (**b**) Four quadrants of enamel topography showing erosion for various times less than 30 min (Image size 1240 × 940 μm). Reprinted with permission from [66]. Copyright 2009 Academy of Dental Materials Published by Elsevier Ltd.

*Streptococcus mutans* is considered a major causative of tooth decay due to its ability to rapidly metabolize carbohydrates such as sucrose, resulting in lactic acid production. Lactic acid causes a decrease in the pH of the oral environment with subsequent demineralization of the tooth enamel. Figure 9 shows the observed topography and surface roughness of a polished dental enamel sample before and after exposure to a solution of citric acid [66]. Typical average roughness values R_a_ and R_RMS_ before etching with citric acid were 21.3 nm and 28.6 nm. Etching with 10% citric acid results in a significantly increased roughness initially, that leveled off at around 1 μm. After 10, 15, and 45 s the roughness values were 200, 300, and 900, respectively. In addition, height measurements can be taken at each interval to quantify demineralization. Enamel height typically falls a few microns in the first 10–15 s in the citric acid. The erosion rate slows to 1 μm/min after about 25 min.

**Figure 10:**
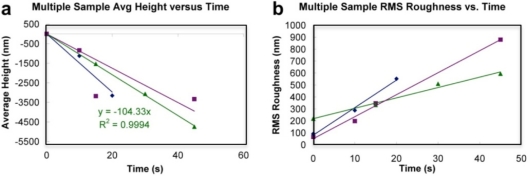
Dental enamel surface height and roughness as metrics to quantify mineral loss. (**a**) Variation in surface height between one minute and 30 minutes. (**b**) Variation in roughness between one minute and 30 minutes (from image size 1240 × 940 μm). After about one minute the RMS roughness plateaus, as does the erosion rate. Data from several samples suggests erosion for less than one minute proceeding rapidly as pits are formed and the smooth enamel surface quickly roughens. Reprinted with permission from [66]. Copyright 2009 Academy of Dental Materials Published by Elsevier Ltd.

Enamel surface develops small pits initially, possibly at structurally weaker enamel regions dissolved by the acid. These smaller pits grow vertically and laterally, coalescing to form larger pits. After significant erosion, there are pits microns deep and wide and spires of tough undissolved enamel. The topography has a relatively larger surface area and the erosion rate correspondingly increases [66]. When the pits coalesce enough they deplete an entire layer, and around this time the system reaches a sort of equilibrium where erosion continues at a roughly constant rate and roughness settles to about 1 micron as shown in Figure 10.

Nanoscale topology and quantitative analysis of dental surfaces are of significant interest. The unique ability of optical profilometry to measure nanometer scale surface properties non-destructively, demonstrates the capacity of this technique to study processes causing morphological changes to the tooth surface itself and dental materials. Conceivably, optical profilometry may even be useful in a dental setting as a profilometer that has the capability to be modified for diagnostic purposes [66].

## 9. Sub-cellular Vesicles as Novel Biomarkers for the Detection of Oral Cancer

**Figure 11:**
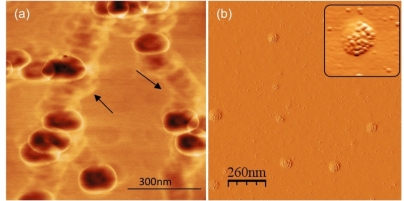
(**a**) Ultrastructure of individual saliva exosomes observed under AFM showing distinct round morphology of exosomes. AM-AFM phase image of aggregated exosomes indicates interconnections (arrows) lacking characteristic phase shift, probably indicate some extra-vesicular protein content. (**b**) Biochemical characterization of exosomes via AFM immunogold imaging. Inset shows 5 nm Au beads marking CD63 receptors on the exosome surface. Reprinted with permission from [70]. Copyright 2010 American Chemical Society.

No other oral diseases are as life threatening as oral and pharyngeal cancer. Oral cancer strikes an estimated 34,360 Americans each year [71]. Worldwide oral cancer is the eleventh most common cancer [72]. Often, oral cancer is preceded by the presence of clinically identifiable premalignant changes. Dental professionals can play a crucial role by identifying these changes during regular once-a-year dental check-up as an effective method for reducing the incidence and mortality of cancer. Saliva meets the demands for inexpensive, noninvasive and easy-to-use diagnostic medium containing proteomic and genomic markers for molecular disease identification [73]. A specialized class of biomarkers found in human saliva that has gained renewed interest is a unique type of sub-100 nm membrane bound secretory vesicles called “exosomes”. Exosomes are secreted by salivary glands epithelium and released into the salivary fluid via exocytosis [74]. Exosomes possess cell type specific membrane and proteins enclosed in a lipid bilayer. Malignancy and other diseases cause elevated exosome secretion and tumor-antigen enrichment of exosomes associated with cancer cells [75,76]. Due to their small size, sensitive and quantitative detection tools are needed for detection and characterization of salivary exosomes. Single vesicle structural and surface molecular details on human saliva exosomes considered as potential non-invasive biomarker resource for oral cancer [77] have been studied recently using AFM [70,78]. Phase images (Figure 11(a)) reveal similar vesicle morphology with diameters of 100 ± 10 nm and an indent in the center of the vesicles [70]. Channel like elongations between exosomes appear without a prominent phase contrast while exosomes show some aggregation without inter-vesicular fusion. Exosomes display a characteristic ring-like tri-lobed structure with one centre appearing as a depression with characteristic phase contrast suggesting role of heterogeneous density and/or viscoelastic image contrast mechanisms. The observed AFM phase contrast of exosomes indicates non-homogenous surface which is tentatively attributable to the presence of proteins and/or mRNA enclosed inside the highly dense lipid membrane [70], consistent with previous proteomic and RNA analysis of saliva and other exosome populations [79]. Single exosomes vesicle ultra-structure, quantitative surface molecular constitution and nanomechanical characteristics of exosomes [70] may be helpful for understanding the patho-physiological role of exosomes in intercellular communication and delivery of genetic components through the extracellular domain.

AFM can sense specific receptors on biological cell interfaces [80,81] such as CD63 receptors on individual exosomes. Sharma *et al.* used quantitative biochemical characterization of exosomes via AFM imaging of bound biofunctionalized gold-beads for highly specific and sensitive detection of CD63 receptor cancer markers [70]. Visualization of labeled exosomes via anti-CD63 and secondary antibody coated gold beads (Figure 11(b)) indicates specific recognition of CD63 molecules. Multiple beads bound to exosomes indicate the presence of multiple CD63 molecules over a single membrane. This was verified by using non-specific primary antibody as control showing no preference for liposome binding. The recognition of single receptor molecules on biological fluid derived exosomes, such as saliva, can potentially detect surface tumor-antigen enriched cancer exosomes, and thereby enable early cancer diagnosis where conventional methods may prove ineffective due to sensitivity limitations [70].

## 10. High-speed AFM: Towards Non-Invasive Nanoscale Measurements in the Dentist’s Surgery

**Figure 12:**
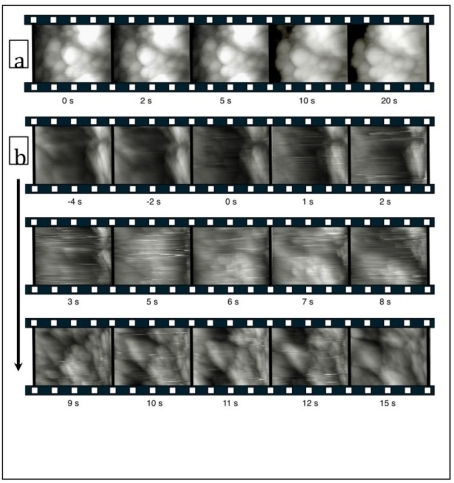
Frames (1.5 μm × 1.5 μm) taken from HS AFM movies of the surface of polished bovine enamel. Film strip (**a**) is a sequence of images record in water; the film strips in (**b**) show a sequence of images recorded before, during, and after the addition of citric acid at pH 3. The time of the addition of the acid is marked as 0 s. Reprinted with permission from [85]. Copyright 2009 International Society of Histology and Cytology.

On the exciting new developments in AFM is the ability to create real time videos of dynamic processes with rates up to 1000 frames per second [82]. Normally AFM takes several minutes to create an image and is too slow to observe high-speed dynamical events, however, recent developments have opened a new avenue to video rate imaging [83,84]. This enables the investigation of how teeth decay on the nanometer scale with millisecond resolution. This, we believe, combined with miniaturization, may open the way to study genetic and other variations in living teeth by *in vivo* oral examination. High-speed atomic force microscopy (HS AFM) in ‘contact’ mode has been applied to image at video rate the surfaces of both calcium hydroxy-apatite samples, which are model artificial dental enamel as well as polished actual bovine dental enamel in both neutral and acidic aqueous solution. The image in each frame of the video suggests the sample was a few micrometers square, and the high-speed scan window was moved across the sample in real time to examine larger areas. HS AFM was shown to dynamically study processes occurring in liquid on the timescale of a few seconds was employed to study dissolution process of both hydroxy apatite and bovine enamel under citric acid solution [85]. Buffered citric acid (pH = 3.0 and 4.0) clearly showed video evidence of surface removal and that this was a spatially heterogeneous process. The movies recorded display rapid dissolution of the bovine enamel in particular. Figure 12 shows frames captures from a movie recorded of the erosion of bovine enamel with and without citric acid in solution [85]. The advantage of lateral and vertical nanometer scale resolution is that, on short time scales, the erosion can be assessed even in situations where the erosion rate is low and would normally require hours to assess by optical microscopy and related micrometer resolution techniques. Furthermore, high frame rates (>10 fps) minimize thermal drifts in the image window and reduce interference from low frequency mechanical noise. This indicates that with sufficient development, AFM may be used as a non invasive technique to assess the effect of treatment and susceptibility to decay as well as measuring biofilm activity in the oral cavity of patients. It also provides a method to quickly screen oral products and the efficacy which when applied to individuals would be useful in personalized dental care procedures by dentists. Therefore, such high speed techniques may find themselves utilized in the dentist’s surgery on a regular basis since the technique involves no radiation or invasive surgical procedures.

## 11. Summary

Nanodentistry is an emerging field with significant potential to yield new generation of technologically advanced clinical tools and devices for oral healthcare. Nanoscale topology and quantitative biomechanical or biophysical analysis of dental surfaces are of significant interest. In particular, using AFM techniques- diseases such as dental caries, tooth hypersensitivity, peridontitis and oral cancer can be quantified based on morphological, biophysical and biochemical nanoscale properties of tooth surface itself and dental materials or oral fluids such as saliva. Based on new understanding of the oral microbial community interactions, there is now interest in approaches that selectively inhibit oral pathogens or modulate the microbial composition of dental plaque to control community based microbial pathogenesis [86]. Future mouthwashes may incorporate smart nano-machines that can identify and explicitly kill pathogenic bacteria without affecting the normal oral flora. Nanoscale characterization of biocompatible implants, tooth sensitivity therapy, plaque management and *in vitro* diagnostics are among the earliest applications of nanotechnology rapidly translating from research to clinical dental environments targeted towards alternative and better approaches to restorative, preventive and cosmetic dental practices. Within the field of restorative dentistry, the tremendous advances in biomaterials nano-characterization and manipulation have led to the current availability of esthetic posterior adhesive restorations and there are now many examples of commercially available products demonstrating the scope of further applications of such technology.

The current generation of AFMs can be integrated with complementary methodologies, including ionic conductance [87], total internal reflection fluorescence (TIRF) [88,89], fluorescence resonance energy transfer (FRET) [90] and other physico-chemical measurements [91], thereby enabling detailed structure-function studies of biofilms, dental surfaces and implants. Rapid quantitative changes in oral surfaces and cell morphology, motion and mechanical rigidity via live-cell interferometry (LCI) [92], can also be combined with the dynamic capability of AFM. Depending on the specific interests and technical requirements, the variety of combination techniques available with AFM would cover both transparent (e.g., dental biofilms) and non-transparent samples such as dental implants and fillings.

As the capability to detect, measure and manipulate surfaces and structures at the nano-dimensional scale progresses, dental applications of nano-enabled and nano-enhanced products and technologies including AFM are bound to rise in the coming years. Indeed, nanotechnology promises to be the critical enabling technology to realize personalized dental care-customized to serve patient needs based on their exact genetic and molecular diagnostics [93]. Future advances are likely through emerging interdisciplinary collaborations and application of new nanotechnology advances to the oral health environment. Finally, the development and miniaturization of AFM as well as recent high speed imaging may one day offer the intriguing possibility to actually perform diagnostic measurements *in vivo* in the dentist’s office similar to the way dental X-rays are routinely taken today.
